# Immunofluorescence Assay and PCR Analysis of *Cryptosporidium* Oocysts and Species From Human Feacal Specimens

**DOI:** 10.5812/jjm.10284

**Published:** 2014-06-01

**Authors:** Mehdi Vejdani, Rezaei Mansour, Yezdan Hamzavi, Sina Vejdani, Naser Nazeri, Ali Michaeli

**Affiliations:** 1 Department of Parasitology and Mycology, Kermanshah University of Medical Sciences, Kermanshah, IR Iran; 2 Department of Statistic and Epidemiology, Kermanshah University of Medical Sciences, Kermanshah, IR Iran; 3 Department of Bioscience, University of Calgary, Alberta, Canada

**Keywords:** *Cryptosporidium parvum*, Fluorescent Antibody Technique, Polymerase Chain Reaction

## Abstract

**Background:**

*Cryptosporidium* spp. is a widespread protozoan parasite involving humans and animals.

**Objectives:**

This study was carried out to evaluate the immunofluorescence assay (IFA) and PCR results for more accurate diagnosis of faecal specimens.

**Patients and Methods:**

Forty six faecal human specimens of *Cryptosporidium* oocysts were examined by PCR and IFA in Calgary, Canada. In statistical analysis, sensitivity and positive predictive value were detected by IFA.

**Results:**

Among 46 faecal samples, 9 (19.6%) were IFA-positive and 10 (21.7%) PCR-positive. Faecal smears of both PCR- and IFA-positive shown that the reproducibility was 90.9% for PCR-DNA and 81.8% for IFA. In Our findings, PCR -DNA showed that diagnosis cryptosporidiosis 2.1% was more sensitive than IFA. Two different oocysts sizes were visualized by IF microscopy which belonged to different species. Furthermore, PCR analysis with primers against the 18S rRNA gene indicated two genotypes of *C. hominis* and *C. parvum*, 500-650 base pairs (bp). In this study, the golden standard was the PCR. In statistical analyses, IFA had positive predictive value (PPV) of 81.8% with 81.8% sensitivity, whereas negative predictive value (NPV) was 1% with 0.97% specificity.

**Conclusions:**

PCR showed more sensitivity than IFA for tracking *Cryptosporidium* oocysts as well as detection of species in faecal human specimens.

## 1. Background

The protozoan parasite *Cryptosporidium* is a major cause of waterborne diarrhea in humans and animals. Tyzzer first described a member of the *Cryptosporidium* genus as an obligate intracellular parasite in mammalians in 1907 (1), but almost after 70 years, the first cases of infections in human were reported (2). The apicomplexan genus *Cryptosporidium* comprises valid species causing intestinal diseases in humans and animals (3, 4). It primarily infects the epithelial cells of gastrointestinal tract, resulting in acute and profuse watery diarrhoea, typically self-limited in immunocompetent individuals, but persistent and potentially life threatening in immunocompromised hosts (5). Among these species, *C. hominis* and *C. parvum* are associated with massive diarrhea outbreaks worldwide, generally caused by exposure to drinking recreational water or direct contact with infected persons through oral-faecal route. These two species have distinct epidemiological cycles, with *C. hominis* infecting mainly humans. *C. parvum,* the most prevalent zoonotic species of the genus *Cryptosporidium,* infects a large number of animals and humans (3).

A recent research has demonstrated that humans and calf are susceptible to infection with at least two distinct, apparently host-adapted genotypes of *Cryptosporidium*. Differentiation between human and bovine genotypes of *C. parvum* is based on the size of the PCR product (6). Two methods of PCR and immunofluorescence assay (IFA) have been discussed between parasitologists for developing PCR in water samples. PCR sensitivity is assessed by the lowest number of oocysts added to the experimental samples, leading to amplification. Comparison of PCR and IFA is complicated due to their different types of data. Nonquantitative PCR yields dichotomous, categorical results (presence or absence of amplifiable DNA), and IFA yields interval data (number of present oocysts). 

Nested-PCR has considerably increased the amplification sensitivity of *Cryptosporidium* DNA, extracted from faecal smears. The use of rapid cycling of real-time PCR has provided such improvements for *C. hominis* and *C. parvum* (7, 8). Inhibitory substances in sediments (such as human acids) affected the PCR performance, leading to false positive-negative PCR results, as indicated by IFA (9). PCR evaluation of *Cryptosporidium* with IFA can be useful and practical for accuracy in PCR-based clinical diagnosis of *Cryptosporidium*.

## 2. Objectives

This study was carried out to compare IFA and PCR assays for more accurate diagnosis of faecal specimens.

## 3. Patients and Methods

Forty six human faecal specimens, *Cryptosporidium-* and *Giardia*-positive, were collected and examined from Chinook regional hospital laboratory, Canada, using IFA and 4,6-diamino-2-phenylindole (DAPI; Sigma-Aldrich, Canada) staining characteristics, then visualized twice preciously in Calgary Provincial Laboratory. These specimens were previously collected from diarrhea patients and preserved by MGL (formalin-ethyl acetate sedimentation) method. To prepare the specimens for testing by monoclonal antibody (mAb)-based IFA (EasyStain; Biotechnology Frontiers, Australia), samples were concentrated using the MGL procedure with centrifugation at 650 × g for 10 minutes to enrich the *Cryptosporidium* oocysts. A few concentrated samples were placed on the fixation slide, with wells installed by the manufacturer; then, the slides were fixed with methanol and allowed to dry at room temperature. 

IFA was performed according to the manufacturer’s instruction, so that mAbs (50 µL) were added to each well, and the slides were incubated in a humidity chamber for 30 minutes at room temperature. The slides were rinsed with phosphate buffered saline (PBS) (pH = 7.5) provided in the kit and placed in a jar containing PBS for 5 minutes. Fluorescein-conjugated EasyStain is an mAb of IgG1 with superior specificity against *Cryptosporidium*. The slides were dried at the room temperature and then mounting medium was added on the slides and they were covered with coverslips. The slides were completely scanned by a fluorescent microscope at 400x. All samples were examined with fluorescein isothiocyanate (FITC) for finding *Cryptosporidium* oocysts specified by the criteria. Epi-fluorescence was used to scan the entire wells for apple-green fluorescence of the oocysts with thick wall, in which brilliant apple-green fluorescing ovoid or spherical objects of 3.2 × 4.2 and 4.2 × 5.2 µm were observed with brightly highlighted edges. Afterwards, the microscope was switched to the UV filter block for DAPI, by which sky-blue nuclei with 1-4-nuclei were exhibited.

The slides were identified and separated for PCR analysis. At first, PBS was added and scraped with plastic scraper. Then, the aliquot was removed by automatic pipettors and freezed at 20ºC for three weeks. Oocysts were purified, followed by PCR amplification (Appliied Biosystems, USA). The DNA was extracted from the oocysts using QIAamp DNA Stool Mini Kit (Applied Biosystems). PCR of 18S rRNA was performed, as previously described by Watanabe et al. (10). Molecular genotyping of *Cryptosporidium* spp. was carried out by nested-PCR–restriction fragment length polymorphism (RFLP) analysis based on the small-subunit (18S) rRNA gene. 

Multiple nested-PCRs were carried out after each DNA extraction, using the PCR conditions and primers described by Xiao et al. (11, 12). A fragment of the 18S rRNA gene was amplified using primer pairs referred to as 18 SiF and 18 SiR. PCR amplification was performed in 50 µL volumes with 1 µL of DNA in 10 x PCR buffer, 10 mmol of each dNTP (Invitrogen Corporation), 10 pmol of each primer, and 1 µL of Taq DNA polymerase (Invitrogen Corporation). The tubes were placed for 30 cycles of 94˚C for three minutes, 94˚C for 45 seconds, 55˚C for 45 minutes, 72˚C for one minute, 72˚C for seven minutes, and at 72˚C for 10 minutes. 

PCR results were detected by agarose gels electrophoresis. The DNA was separated using 2.0% agarose gels, run in tris-acetate (TAE) buffer (0.04 mM TAE, 0.001 mM EDTA, pH = 8.0) at 100 V cm-1 for 95 minutes, stained in ethidium bromide solution (0.5 ug/mL), and visualized with a UV transilluminator. All the products, positive by nested PCR, were digested with SspI, VspI, and Ddel restriction enzymes (11-13), fractionated by 2% agarose gel electrophoresis, and visualized by ethidium bromide staining.

## 4. Results

Eleven of 46 fecal fixed slides were provided for detecting *Cryptosporidium* oocysts by IFA and PCR/RLFP. Of 46 IFA slides, 9 (19.6%) were visualized by IF microscope. They were recognized as positive for *Cryptosporidium* oocysts, with the thick walls and brilliant apple-green walls of 3.2 × 4.2 and 4.2 × 5.2 µm, respectively. Thirty seven slides were enumerated under the IF microscope and were negative for *Cryptosporidium* oocysts. Our findings showed a numerical variation between < 8 to 500 oocysts (Table 1). Ten (21.7%) of 46 slide were positive by PCR (Table 1). In PCR-positive samples, performed by DNA recovery, the PCR reproducibility was determined 90.9% and IFA-oocysts were visualized with 81.8% sensitivity (calculated as follows: [number of true positives/(number of true positives × number of false negatives)] × 100, and there were 80% correlation between PCR and IFA, or between a positive amplification of *Cryptosporidium* PCR-bp target and IFA-oocysts for detecting *cryptosporidium* spp in slides of fecal human specimens).

**Figure 1.**
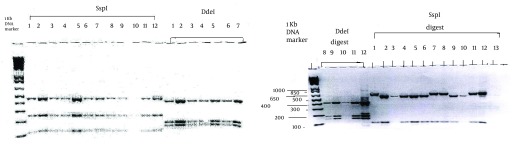
PCR Products Digested With *SspI, VspI and DdeI* Restriction Enzymes, Displayed in Panels A, B and *C*: lanes 1, 8 and 11; *C. parvum* digested with *SspI, DdeI and VspI*. Lanes 2-6 and 9-10; *C. hominis* digested with *SspI, DdeI and VspI*. Lanes 7 and 12 with 650-bp lambda DNA; *C. parvum* positive control. Lane 13; negative control.

One (2.2%) of 46 slides revealed 57 oocysts by IFA, which appeared to be a discordant result, whereas their PCRs were negative. Furthermore, 2 (4.3%) slides were IFA-oocyst-negative, while their PCR results were positive (Table 1). The PCR products were related to the presence of 500-650-bp amplicons of the *Cryptosporidium* oocysts 18S rRNA genes in 2% agarose gel electrophoresis. The *Cryptosporidium* DNA was confirmed with *Cryptosporidium* primer pairs. PCR-positive fecal human specimens were related to *C. hominis* and *C. parvum* characteristics (Figure 1). In statistical analysis, IFA had 81.8% sensitivity and positive predictive value (PPV) of 81.8 %, whereas negative predictive value (NPV) was 1% and specificity was 0.97%.

**Table 1.** Comparison Between IFA and PCR Results of 46 Human Fecal Specimens for Detection of *Cryptosporidium* Oocysts ^a^

Enumerated Oocyst	IFA Specimens	IFA Brilliant Apple-Green Oocyst as Positive Control	PCR Specimens	PCR of *Cryptosporidium parvum* as Positive Control	PCR Master Mix as Negative Control	PCR Negative Control
**-**	-	+	+	+	-	-
**-**	-	+	+	+	-	-
**< 8**	+	+	+	+	-	-
**41**	+	+	+	+	-	-
**53**	+	+	+	+	-	-
**57**	+	+	+	+	-	-
**65**	+	+	+	+	-	-
**81**	+	+	+	+	-	-
**85**	+	+	+	+	-	-
**265**	+	+	+	+	-	-
**> 500**	+	+	+	+	-	-

^a^ Abbreviations: IFA, immunofluorescence Assay; PCR, polymerase chain reaction.

## 5. Discussion

In diagnostic laboratories, *Cryptosporidium* oocysts are reported based on the results of one of the two different microscopic methods, acid-fast stain or IFA (2). In the two past decades, the PCR/RFLP technique has developed to detect and distinguish *C. hominis* from *C. parvum*. Nested-PCR considerably increases the amplification sensitivity of *Cryptosporidium* DNA extracted from water, whole faces and fecal smears. Further improvements in molecular detection of parasites would reduce the amplification time and their PCR products (8). In the present study, we performed PCR/RFLP and IFA for human faecal samples. Our study showed that PCR/RFLP was 2.1% more sensitive than IFA.

*Cryptosporidium* oocysts PCR has been a useful tool for detecting pathogens in environmental samples, offering more sensitive and specific detection (14). Some researchers have described that a common drawback of PCR is lack of viability determination, which is in contrast with the currently accepted IFA (5). This study revealed that 10 (90.9%) of 46 smears were PCR-positive and 9 (81.8%) were IFA-positive. Although, In statistical analysis, IFA had 81.8% sensitivity with PPV of 81.8%, but the specificity was as low as 0.97%. However, sensitivity of IFA has a very high importance. Although, other studies explained that PCR analysis identified 100% sensitivity, while microscopic procedures (acid-fast stain) had 83.7% sensitivity (6).

PCR amplification can be an obvious choice for improving the *Cryptosporidium* detection from feces (6). In one study, PCR was compared with IF for detection of *Cryptosporidium*, and due to a number of problems, including inhibition, PCR was no more sensitive than IF (6, 15). However, our findings showed that IFA is less sensitive than PCR, but easy and inexpensive. On the other hand, knowledge and experience of the microscopist are critical in scanning the oocysts as considerable issues in IFA, whereas inhibitors and contaminant agents are effective in PCR assay. Other researchers compared PCR with both auramine phenol and IF staining in bovine feces, and reported that the immunomagnetic separation used to purify the oocysts was more sensitive than conventional techniques (6, 16).

In this study, PCR was more accurate and diagnosed more human faecal oocysts. Some studies illustrated that while a simple inexpensive morphology-based identification can be used to detect *Cryptosporidium* in stool samples, only molecular approaches guarantee the identification to the species level (3). However, other studies found discrepancies comparing PCR and IFA for *Cryptosporidium* detection (6, 15). Our study indicated a difference between PCR and IFA, as PCR was more sensitive than IFA. In addition, the reproducibility of DNA extracted from faecal smears was 90.9%. Some studies have recovered DNA from faecal smears, about 85.3% that may be occurred a co-extraction of inhibitors of the PCR or of DNA from the faecal microflora (8).

Since oocysts were rarely detected via IFA, it lacks sensitivity (2). on the other hand, some researcher suggested that they could be IFA staining method is unexpectedly high (17). One of the faecal smears was IFA-positive; in contrast, its PCR was negative. It is possible that there have been PCR inhibitors in faecal specimens. A study revealed that faecal constituents such as bilirubin, bile salts and complex polysaccharides inhibit PCR, even at low concentrations (13). Human acids affect PCR, as DNA may not be replicated in some or all aliquots from a single sample. Based on PCR genotyping, *C. parvum* bovine genotype and *C. hominis* from human faecal specimens were detected as 500 and 650 bp. Among *Cryptosporidium* species, *C. parvum* and *C. hominis* are associated with massive outbreaks worldwide (9).

In this study, *C. hominis*, infecting mainly humans, and *C. parvum* as the most prevalent zoonotic species of the genus *Cryptosporidium,* can be involved with a large number of animal species and humans. Our result confirmed that PCR using 18S rRNA gene primers, could provide more sensitivity than IFA. PCR-based analyses using 18S rRNA gene primers have been useful for genotyping and IFA has been beneficial for laboratory and environmental samples diagnoses. However, important usefulness factors of IFA include being time consuming and the necessity for an expert personnel. Most of the investigations suggested PCR as the most effective purification method for *C. parvum* oocyst detection (18). Findings of this study indicated that PCR was more accurate than IF antibody test (IFAT), but unable to detect more less than one oocyst in faecal specimens, whereas IFAT can be exhibited by an expert parasitologist and with experienced workers to numerate the oocysts under the microscope.

PCR has been a molecular approach for finding the *Cryptosporidium* oocysts DNAs. PCR has shown more sensitivity than IFA for tracking *Cryptosporidium* oocysts and detecting its genotypes in faecal human specimens. Likewise, IFA has been suitable and faecal specimens should be examined by an experienced parasitologist. PCR was the golden standard in our study.

## References

[1] Tyzzer EE (1907). A sporozoan found in the peptic glands of the common mouse.. Exp Biol Med..

[2] Ignatius R, Eisenblatter M, Regnath T, Mansmann U, Futh U, Hahn H (1997). Efficacy of different methods for detection of low Cryptosporidium parvum oocyst numbers or antigen concentrations in stool specimens.. Eur J Clin Microbiol Infect Dis..

[3] Bandyopadhyay K, Kellar KL, Moura I, Casaqui Carollo MC, Graczyk TK, Slemenda S (2007). Rapid microsphere assay for identification of cryptosporidium hominis and cryptosporidium parvum in stool and environmental samples.. J Clin Microbiol..

[4] Davies CM, Kaucner C, Deere D, Ashbolt NJ (2003). Recovery and enumeration of Cryptosporidium parvum from animal fecal matrices.. Appl Environ Microbiol..

[5] Liu J, Deng M, Lancto CA, Abrahamsen MS, Rutherford MS, Enomoto S (2009). Biphasic modulation of apoptotic pathways in Cryptosporidium parvum-infected human intestinal epithelial cells.. Infect Immun..

[6] Morgan UM, Pallant L, Dwyer BW, Forbes DA, Rich G, Thompson RC (1998). Comparison of PCR and microscopy for detection of Cryptosporidium parvum in human fecal specimens: clinical trial.. J Clin Microbiol..

[7] Amar C, Pedraza-Diaz S, McLauchlin J (2001). Extraction and genotyping of Cryptosporidium parvum DNA from fecal smears on glass slides stained conventionally for direct microscope examination.. J Clin Microbiol..

[8] Amar CF, Dear PH, McLauchlin J (2004). Detection and identification by real time PCR/RFLP analyses of Cryptosporidium species from human faeces.. Lett Appl Microbiol..

[9] Walker MJ, Montemagno C, Bryant JC, Ghiorse WC (1998). Method detection limits of PCR and immunofluorescence assay for Cryptosporidium parvum in soil.. Appl Environ Microbiol..

[10] Watanabe Y, Yang CH, Ooi HK (2005). Cryptosporidium infection in livestock and first identification of Cryptosporidium parvum genotype in cattle feces in Taiwan.. Parasitol Res..

[11] Xiao L, Alderisio K, Limor J, Royer M, Lal AA (2000). Identification of species and sources of Cryptosporidium oocysts in storm waters with a small-subunit rRNA-based diagnostic and genotyping tool.. Appl Environ Microbiol..

[12] Xiao L, Morgan UM, Limor J, Escalante A, Arrowood M, Shulaw W (1999). Genetic diversity within Cryptosporidium parvum and related Cryptosporidium species.. Appl Environ Microbiol..

[13] Ruecker NJ, Braithwaite SL, Topp E, Edge T, Lapen DR, Wilkes G (2007). Tracking host sources of Cryptosporidium spp. in raw water for improved health risk assessment.. Appl Environ Microbiol..

[14] Mayer CL, Palmer CJ (1996). Evaluation of PCR, nested PCR, and fluorescent antibodies for detection of Giardia and Cryptosporidium species in wastewater.. Appl Environ Microbiol..

[15] Johnson DW, Pieniazek NJ, Griffin DW, Misener L, Rose JB (1995). Development of a PCR protocol for sensitive detection of Cryptosporidium oocysts in water samples.. Appl Environ Microbiol..

[16] Webster KA, Smith HV, Giles M, Dawson L, Robertson LJ (1996). Detection of Cryptosporidium parvum oocysts in faeces: comparison of conventional coproscopical methods and the polymerase chain reaction.. Vet Parasitol..

[17] Weber R, Bryan RT, Juranek DD (1992). Improved stool concentration procedure for detection of Cryptosporidium oocysts in fecal specimens.. J Clin Microbiol..

[18] Kar S, Gawlowska S, Daugschies A, Bangoura B (2011). Quantitative comparison of different purification and detection methods for Cryptosporidium parvum oocysts.. Vet Parasitol..

